# TGF‐β Blockade With SB525334 Enhances B7‐H3 CAR‐γδT Cell Efficacy Against Glioblastoma

**DOI:** 10.1111/jcmm.71089

**Published:** 2026-03-25

**Authors:** Yang Zhu, Zijian Han, Zijing Zhou, Yinqiang Sui, Yang Zhang, Zuoyu Jiang, Qinzhi E, Xuewen Zhang, Weichao Wang, Yingbo Hou, Jiaming Du, Yi Tang, Hanmiao Dong, Yeyang Xu, Ruoyu Sun, Chenyang Li, Xuetao Li, Yulun Huang

**Affiliations:** ^1^ The Fourth Affiliated Hospital of Soochow University Suzhou China; ^2^ Unicet Biotech Beijing China

**Keywords:** CAR‐γδT cell therapy, combination therapy, glioblastoma (GBM), SB525334, TGF‐β signalling, tumour microenvironment (TME)

## Abstract

Transforming growth factor‐beta (TGF‐β) signalling promotes glioblastoma (GBM) immunosuppression and therapy resistance. Although CAR‐γδT therapy has shown promising efficacy in hematologic malignancies, its application to solid tumours—particularly GBM—is impeded by multiple factors, including a highly immunosuppressive tumour microenvironment, inefficient T‐cell infiltration, T‐cell exhaustion driven by inhibitory pathways such as TGF‐β, and antigen heterogeneity. Bioinformatics analyses further corroborate that TGFB1 is significantly upregulated in GBM and correlates with poor prognosis, highlighting TGF‐β as a pivotal mediator of therapeutic resistance. This study aimed to evaluate whether SB525334, a selective TGF‐β receptor inhibitor, can enhance the efficacy and persistence of CAR‐γδT cells in GBM. We demonstrate that SB525334 selectively enhances CAR‐γδT cell function and antitumour efficacy by alleviating TGF‐β–mediated immunosuppression and T‐cell exhaustion while promoting immune activation within the GBM microenvironment. The combination treatment significantly reduced tumour cell viability (approximately 40%–50% residual viability) compared to CAR‐γδT therapy alone (70%–85% residual viability), indicating a clear synergistic effect. Unlike broad‐spectrum inhibitors, SB525334 sustains CAR‐γδT proliferative capacity and effector function under chronic antigen stimulation, remodels the immunosuppressive tumour microenvironment, and promotes a pro‐inflammatory immune signature. Our findings establish a combination strategy to overcome immunosuppressive barriers that limit current CAR‐γδT therapies in solid tumours. By maintaining T‐cell function and remodelling the tumour immune microenvironment, TGF‐β1 small‐molecule inhibitors show potential as translational adjuvants to broaden the clinical applicability of CAR‐γδT cell immunotherapy.

## 1 Introduction

Glioblastoma (GBM) is the most aggressive primary brain malignancy in adults, characterised by inevitable recurrence and a median survival of less than 18 months despite maximal therapy, including surgical resection, radiotherapy and temozolomide chemotherapy [1]. This profound therapeutic resistance arises from the heterogeneous molecular landscape of GBM and a profoundly immunosuppressive tumour microenvironment (TME), which together pose significant barriers to therapeutic advances [2, 3]. Immunotherapeutic approaches, particularly Chimeric Antigen Receptor γδT (CAR‐γδT) cell therapy, have demonstrated remarkable success in hematologic malignancies [4]. However, their efficacy against solid tumours remains severely limited by the GBM TME, which restricts T‐cell infiltration, promotes T‐cell exhaustion and facilitates immune evasion [5, 6, 7].

Central to this immunosuppressive axis is the transforming growth factor beta (TGF‐β) signalling pathway, which undergoes pathological activation in GBM [8]. TGF‐β exhibits a dual oncogenic role: while functioning as a tumour suppressor in normal cells, it becomes a potent driver of tumour progression, maintenance of stemness, epithelial–mesenchymal transition (EMT), angiogenesis, and, critically, systemic immunosuppression [9]. TGF‐β directly inhibits T‐cell proliferation and cytotoxic function while promoting regulatory T‐cell (Treg) differentiation, thereby creating an immunologic barrier that impairs both endogenous immunity and adoptive cellular therapies [10, 11].

Despite growing interest in targeting the TGF‐β pathway to enhance CAR‐γδT therapy in GBM, critical knowledge gaps remain. First, although prior TGF‐β inhibitors (e.g., LY2157299) effectively block downstream signalling [12], they often cause undesirable suppression of CAR‐γδT proliferation, highlighting the need for agents that selectively preserve CAR‐γδT fitness while exerting robust tumour cytotoxicity. Second, although numerous studies report in vitro enhancement of CAR‐γδT function through TGF‐β inhibition [13], few systematically examine how such inhibition remodels the broader GBM immune microenvironment—particularly effects on Treg expansion, myeloid‐derived suppressor cell (MDSC) infiltration, and spatial reconfiguration of immune subsets. Furthermore, no study has definitively addressed the impact of treatment sequencing—specifically, whether preconditioning with TGF‐β inhibition before CAR‐γδT infusion outperforms concurrent administration—a variable that critically influences translational efficacy [14].

We hypothesized that reversing TGF‐β signalling would enhance the tumour‐killing efficacy of CAR‐γδT cells and delay their exhaustion. In this study, we used SB525334, a highly potent and selective small‐molecule inhibitor targeting the kinase domain of TGF‐β receptor I (ALK5). Compared with first‐generation TGF‐β inhibitors such as LY2157299 (IC₅₀ for ALK5 = 130 nM), we selected SB525334 for superior selectivity and mechanistic advantages. Whereas agents such as LY2157299 exhibit broad inhibitory activity against multiple TGF‐β isoforms, posing risks of nonspecific immunosuppression and cardiovascular toxicity, SB525334 demonstrates marked selectivity, with substantially higher affinity for ALK5 than for other closely related kinases [15]. SB525334 binds reversibly to the ATP‐binding pocket of ALK5, competitively inhibiting ATP binding and thereby preventing receptor autophosphorylation and activation. This inhibitor exhibits an exceptionally low half‐maximal inhibitory concentration (IC50) for ALK5, typically in the nanomolar (nM) range (approximately 14.3 nM as reported in the literature) [16]. Such a low IC50 value indicates that only small concentrations of the compound are needed to effectively inhibit receptor activity, which reduces off‐target effects and offers a wider therapeutic window compared with nonselective inhibitors [17]. These properties make it an ideal candidate for combination immunotherapy in the treatment of central nervous system malignancies.

To mitigate antigenic heterogeneity and antigen escape—a major limitation of CAR therapies in GBM—we targeted B7‐H3 (CD276), a pan‐cancer antigen consistently overexpressed in GBM and associated with poor prognosis. B7‐H3 demonstrates homogeneous expression across GBM subtypes and minimal expression in normal brain tissue, thereby providing a robust target for CAR‐γδT therapy while reducing the likelihood of antigen escape [18, 19].

## 2 Results

### 2.1 Bioinformatics Analysis of TGFB1 Expression and Prognosis

**FIGURE 1:**
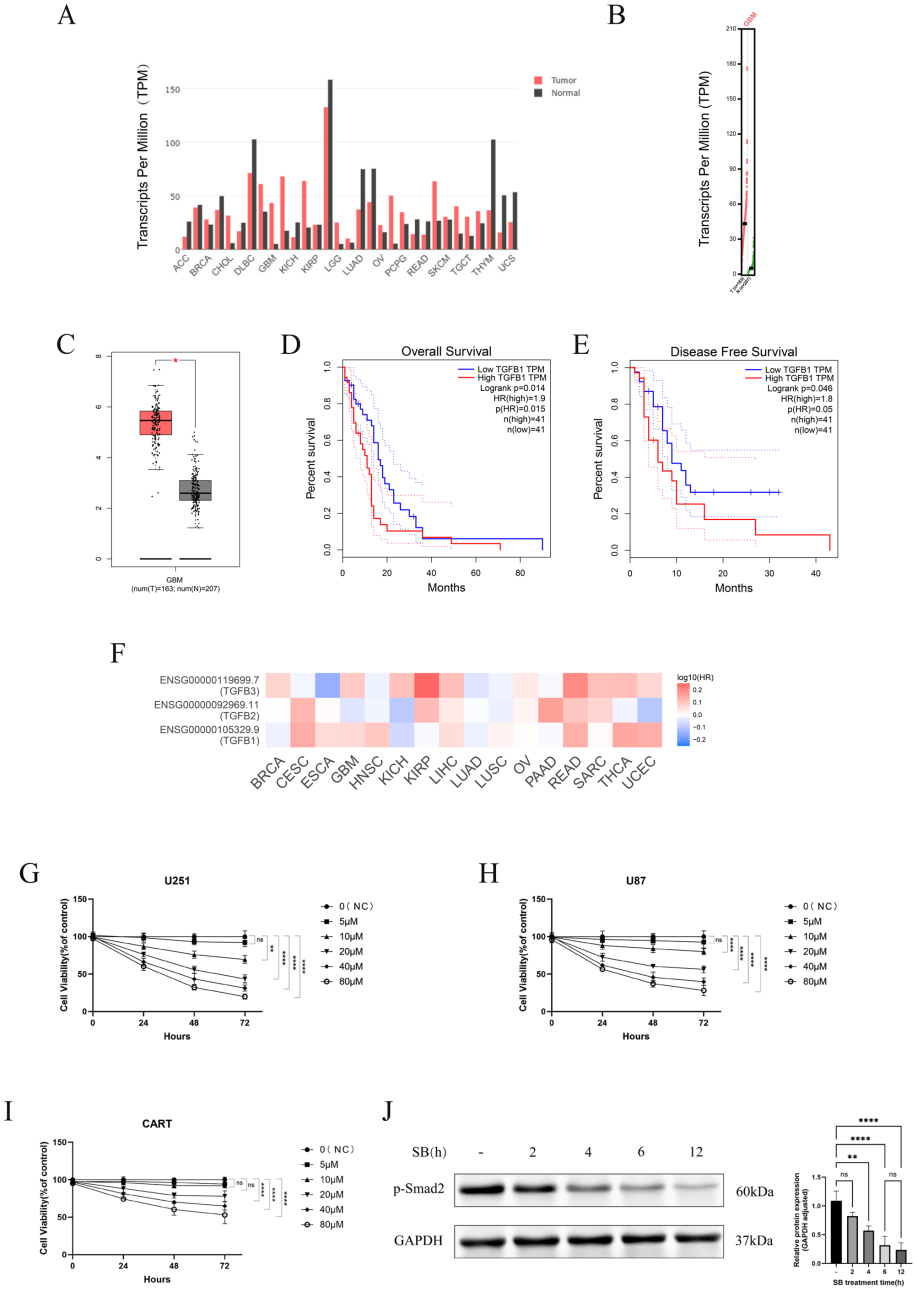
(A) Gene expression profile across solid tumour samples and paired normal tissues. (B) Correlation analysis of TGFB1 expression in GBM. (C) Differential expression of TGFB1 between normal tissues and glioblastoma tissues. (D, E) Survival prognosis analysis following TGFB1 overexpression. (F) Prognostic heatmap of the TGFB family across various cancers. (G, H, I) Cell viability (expressed as percentage of control) of U251, U87 and CAR‐γδT cells treated with different concentrations of SB525334 (0, 5, 10, 20, 40, 80 μM) at various time points (0, 24, 48, 72 h). Mean ± SD, *n* = 4. (J) Expression of p‐SMAD2 in U251 cells treated with TGF‐β (5 ng/mL) with or without SB525334 (5 μM) for 2, 4, 6 and 12 h. Mean ± SD, *n* = 3. Two‐way ANOVA analysis was used in G‐I. One‐way ANOVA analysis was used in J. **p < 0.01, ***p < 0.001, ****p < 0.0001, ns, not significant.

To evaluate the dysregulation of TGFB1 expression across various solid tumours, we analysed data from the GEPIA database and performed validation in an independent glioblastoma (GBM) cohort. The glossary of tumour type abbreviations is provided (Table S1). Our results revealed that TGFB1 dysregulation is a common phenomenon. TPM expression analysis demonstrated significantly higher TGFB1 levels in tumour tissues of multiple solid cancers, including GBM, compared to normal tissues (Figure 1A,B). Box‐plot validation further confirmed a markedly elevated median TGFB1 expression in GBM tumour tissues (tumour: *n* = 163; normal controls: *n* = 207; *p* < 0.001) (Figure 1C), supporting its potential role in GBM pathogenesis.

Survival analysis indicated that median overall survival was significantly shorter in the high TGFB1 expression group (*n* = 41) than in the low‐expression group (*n* = 41; log‐rank test, *p* = 0.014), with a 1.9‐fold increased risk of death (hazard ratios (HR) = 1.9, *p* = 0.015) (Figure 1D). Patients with high TGFB1 expression also had reduced progression‐free survival (log‐rank test, *p* = 0.046; HR = 1.8, *p* = 0.05), confirming TGFB1 as an independent predictor of poor prognosis in GBM (Figure 1E).

A prognostic heat map of the TGFB family revealed differential associations: high expression of TGFB1 (ENSG00000105329.9) correlated with poor prognosis (log_10_[HR] = 0.1–0.2; HR = 1.26–1.58), whereas TGFB2 (ENSG00000092969.11) was associated with improved survival (log_10_[HR] = −0.1; HR = 0.79). TGFB3 (ENSG00000119699.7) showed no significant prognostic association (log_10_[HR] = 0), highlighting the dominant role of TGFB1 in driving unfavourable outcomes in GBM (Figure 1F).

### 2.2 Exploration of the Optimal Administration Concentration of SB525334

To investigate the cytotoxic effects of the TGF‐β receptor inhibitor SB525334 on glioblastoma cell lines, we assessed the viability of U251 and U87 cells treated with various concentrations of SB525334 (0, 5, 10, 20, 40 and 80 μM) at different time points (24, 48 and 72 h), with viability normalised to the negative control. Results showed that cell viability decreased significantly with increasing concentration and longer exposure in both cell lines (Figure 1G,H). High concentrations (e.g., 40 and 80 μM) and longer durations (e.g., 48 and 72 h) produced statistically significant reductions, indicating that SB525334 effectively inhibits glioblastoma cell survival at high concentrations, with minimal effects at low concentrations (≤ 5 μM).

To examine the impact of SB525334 on CAR‐γδT cell viability, CAR‐γδT cells were co‐cultured with SB525334 at concentrations of 0, 5, 10, 20, 40 and 80 μM, and viability was assessed at 0, 24, 48, and 72 h. At low concentrations (≤ 10 μM), no significant change in viability was observed over time (ns), whereas at a high concentration (80 μM), viability decreased significantly with prolonged exposure (Figure 1I). These findings indicate that SB525334 has minimal impact on CAR‐γδT cell viability at low concentrations (≤ 10 μM).

Based on the above experiments, a concentration of 5 μM SB525334 was identified that showed no significant impact on the viability of either glioblastoma cells or CAR‐γδT cells.

To determine whether a low concentration of SB525334 (5 μM) blocks TGF‐β signalling, we assessed the phosphorylation level of Smad2 by Western blot analysis. U251 cell lysates were collected after co‐treatment with TGF‐β1 and 5 μM SB525334 for 2, 4, 6 and 12 h. Compared to the control group, the p‐Smad2 signal was significantly reduced in the 6‐h treatment group. Furthermore, no significant difference was observed between the signals at 6 and 12 h, indicating that a 6‐h treatment with 5 μM SB525334 is sufficient to effectively inhibit TGF‐β signalling transduction (Figure 1J).

### 2.3 Anti‐Glioma Effects of SB525334 Combined With CAR‐γδT Cells In Vitro

**FIGURE 2:**
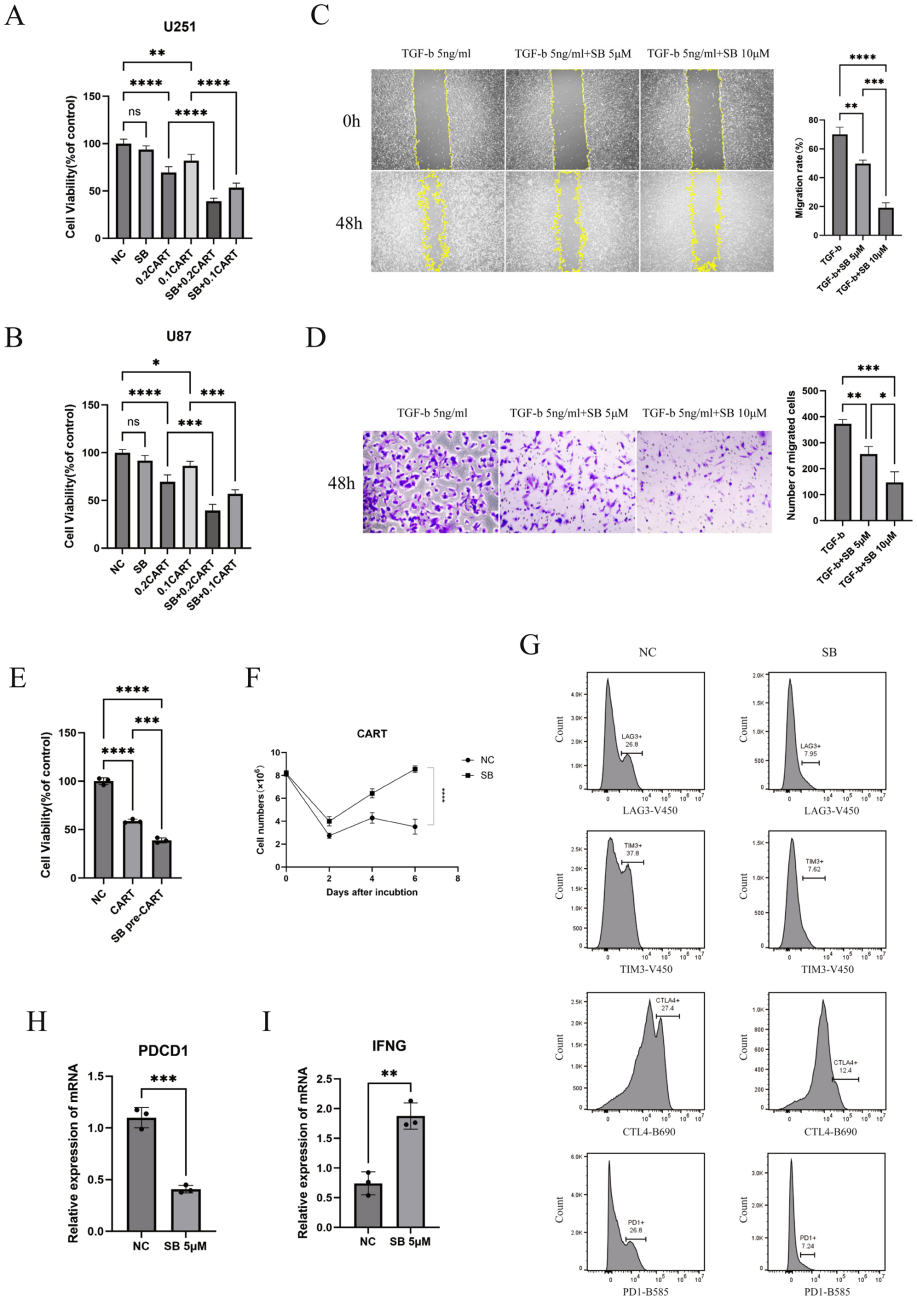
Anti‐glioma effects of SB525334 combined with CAR‐γδT cells at the in vitro level. (A, B) Cell viability assay performed after 48 h of TGF‐β (5 ng/mL) exposure using U251 and U87 cells. NC, negative control; SB, treated with 5 μM SB525334; 0.2 CART, effector‐to‐target ratio 1:5; 0.1 CART, E:T ratio 1:10. Mean ± SD, *n* = 3. (C, D) Effects of SB525334 on glioma cell migration assessed by scratch wound and Transwell assays. Mean ± SD, *n* = 3. (E) In the exhaustion and functional persistence assay, CAR‐γδT cells were collected at the conclusion of the final round of stimulation, followed by assessment of their tumour‐killing ability. Mean ± SD, *n* = 3. (F) Changes in CAR‐γδT cell numbers after each round of antigen stimulation. Mean ± SD, *n* = 3. (G) Surface expression of exhaustion markers (PD1, LAG3, TIM3, CTLA4) analysed by flow cytometry. (H, I) qPCR analysis of the exhaustion marker gene PDCD1 and the functional marker gene IFNG. Mean ± SD, *n* = 3. One‐way ANOVA analysis was used in (A–E). Two‐way ANOVA The unpaired StudentThe unpaired Student (F). The unpaired Student *t*‐test was used in (H) and (I). *p < 0.05, **p < 0.01, ***p < 0.001, ****p < 0.0001, ns, not significant.

To evaluate the effects of the TGF‐β receptor inhibitor SB525334 combined with CAR‐γδT cells on glioblastoma cell viability, a 48 h cell viability assay was performed under TGF‐β1 exposure using U251 and U87 cell lines. In U251 cells, compared with the Negative control (NC) group, SB525334 alone (5 μM) had minimal impact on tumour cell viability. Treatment with 0.2 CAR T (effector‐to‐target ratio 0.2:1) alone reduced viability to approximately 70%, whereas 0.1 CAR T (E:T 0.1:1) alone decreased viability to about 85%. In contrast, the combination of SB525334 (5 μM) with 0.2 CAR T further reduced viability to approximately 40%, and its combination with 0.1 CART lowered viability to around 50%. Statistical analysis indicated a synergistic interaction between the treatments (Figure 2A). A similar trend was observed in U87 cells, further supporting a synergistic mechanism (Figure 2B).

SB525334 inhibited TGF‐β1‐induced migration of U251 cells, as shown by scratch and Transwell assays (Figure 2C,D).

To investigate the effect of SB525334 on CAR‐γδT cell exhaustion, a multiple‐round antigen stimulation assay was conducted. A total of 8 × 10^6^ CAR‐γδT cells were co‐cultured with tumour cells at a 2:1 effector‐to‐target ratio under 5 ng/mL TGF‐β1. Tumour cells were added to the co‐culture system every 2 days at the same E:T ratio. The combination group consistently received 5 μM SB525334. In the final round (third round), CAR‐γδT cells collected from the combination group reduced tumour cell viability to approximately 38%, compared with 57% in the CAR‐γδT–alone group (NC, 100%) (Figure 2E). CAR‐γδT cell counts after each stimulation round showed that the combination group better sustained CAR‐γδT proliferation under repeated antigen exposure (Figure 2F). By the third round, the cell count in the SB525334‐treated group was approximately 2.3 times higher than in the NC group. Furthermore, flow cytometry showed that SB525334 reduced exhaustion markers (LAG3, TIM3, CTLA4, PD1) (Figure 2G), and qPCR analysis indicated decreased PDCD1 expression and increased IFNG levels (Figure 2H,I), confirming that SB525334 helps maintain CAR‐γδT cell function and suppress exhaustion.

### 2.4 Anti‐Glioma Effects of SB525334 Combined With CAR‐γδT Cells In Vivo

#### 2.4.1 SB525334 Potentiates the Tumour Eradication Efficacy of CAR‐γδT Cells in Nude Mouse CDX Models

**FIGURE 3:**
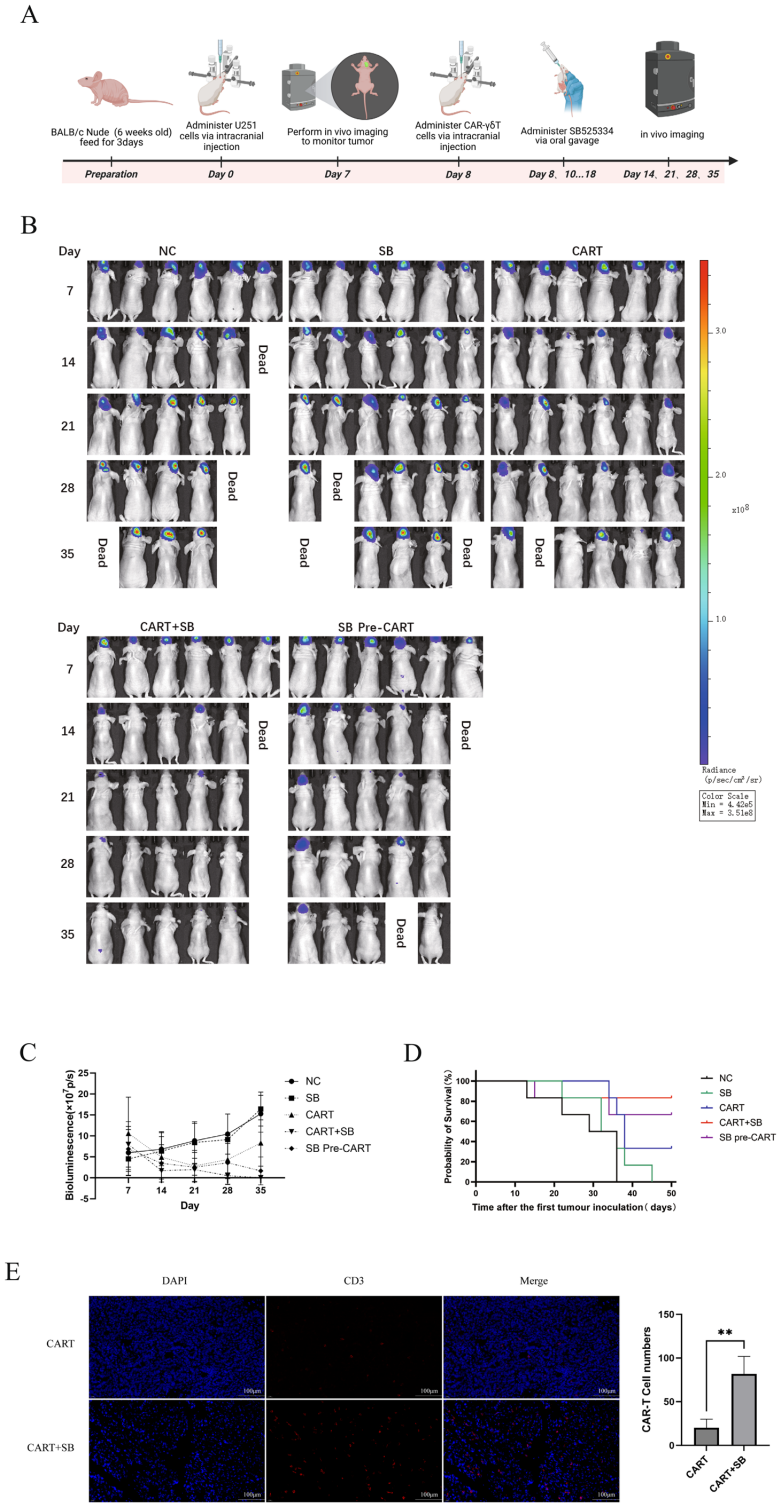
(A) Schematic diagram of the in vivo experimental design using a CDX model. (B, C) Tumour growth monitored by in vivo imaging on days 7, 14, 21, 28 and 35 after inoculation in nude mice, with dynamic changes in bioluminescence intensity recorded. (D) Survival time of mice in each group. (E) Residual CAR‐γδT cells in the brains of mice from the CART group and the combination group on day 14 after tumour inoculation (Representative immunofluorescence staining of mouse brain paraffin sections at 40× magnification). Mean ± SD, *n* = 3. The unpaired Student *t*‐test was used in E. **p < 0.01.

To evaluate the anti‐glioma efficacy of SB525334 combined with CAR‐γδT cells in vivo, a nude mouse model was established. Mice were randomly assigned to five groups (NC, SB, CAR T, CAR T+SB, SB Pre–CAR T). Tumour growth was monitored by in vivo imaging on days 7, 14, 21, 28 and 35 after implantation (Figure 3B). Quantitative analysis of bioluminescence imaging demonstrated that compared with the NC and SB525334‐alone groups, which showed rapid tumour growth and high mortality, the CAR T‐alone group exhibited partial tumour suppression but signs of recurrence. In contrast, the CAR T+SB and SB Pre‐CAR T groups showed significant tumour regression, improved survival and some cures (Figure 3C). Kaplan–Meier survival curves indicated higher survival rates in the CAR T+SB and SB Pre‐CAR T groups, suggesting that SB525334 enhances the antitumour effect of CAR‐γδT cells and delays their exhaustion (Figure 3D). Moreover, residual CAR‐γδT cells in the brains of the CAR T+SB group were notably more abundant than in the CAR T–alone group on day 14 (6 days after CAR‐γδT injection) (Figure 3E).

#### 2.4.2 SB525334 Remodels the Tumour Microenvironment in Syngeneic C57BL/6 Mouse Glioma Models

**FIGURE 4:**
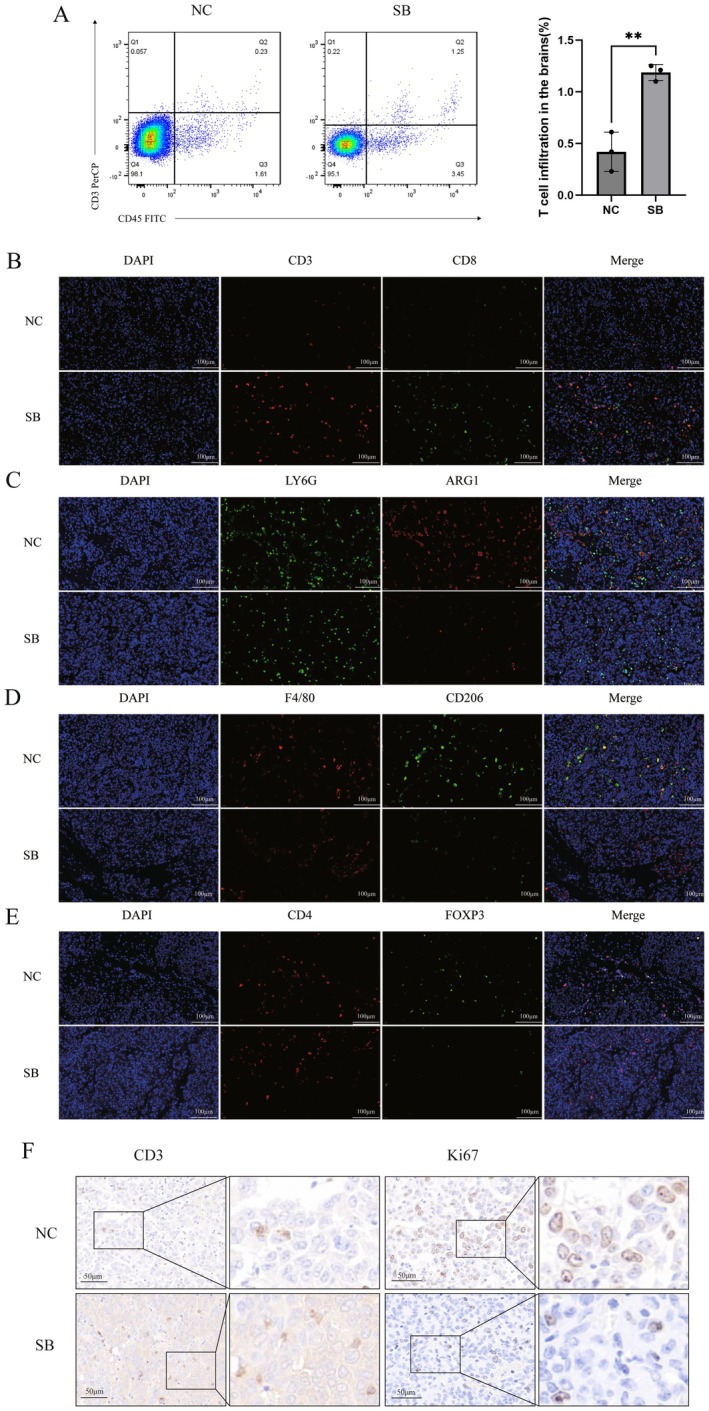
(A) Quantification by flow cytometry of T cell infiltration in the brains of C57BL/6 mice after six rounds of SB525334 gavage (10 mg/kg) administered every other day. Mean ± SD, *n* = 3. (B–E) Representative immunofluorescence staining of mouse brain paraffin sections at 40× magnification. (F) Representative immunohistochemistry staining of mouse brain paraffin sections at 20× magnification. The unpaired Student *t*‐test was used in A. **p < 0.01.

To investigate the effect of SB525334 on the glioma microenvironment, we employed a syngeneic glioblastoma (GBM) model in C57BL/6 mice by inoculating GL261 cells. Mice were divided into a negative control (NC) group and an SB525334‐treated group. Following six rounds of SB525334 administration via oral gavage, brain tissues were collected and analysed by flow cytometry. The results demonstrated a significant increase in T‐cell infiltration in the SB525334‐treated group compared to the NC group, indicating that SB525334 promotes T‐cell infiltration and supports remodelling of the immune microenvironment (Figure 4A).

We further assessed the infiltration of key immune cell populations in glioma tissues after SB525334 treatment using immunofluorescence staining. Compared to the NC group, SB525334 treatment markedly enhanced the infiltration of T cells, particularly CD8^+^ T cells, within the tumour microenvironment (Figure 4B). Concurrently, the treatment reduced the numbers of granulocytic myeloid‐derived suppressor cells (G‐MDSC, LY6G^+^ARG1^+^), M2‐type tumour‐associated macrophages (M2‐TAM, F4/80^+^ARG1^+^), and regulatory T cells (Treg, CD4^+^FOXP3^+^) (Figure 4C–E). These findings suggest that SB525334 reshapes the glioma immune microenvironment by promoting the infiltration of anti‐tumour T cells while suppressing various immunosuppressive cell populations.

Immunohistochemical staining for CD3 and Ki67 further revealed that, relative to the NC group, the SB525334 group exhibited increased CD3 signalling and decreased Ki67 signalling. This indicates enhanced T‐cell infiltration concomitant with the inhibition of tumour cell proliferation (Figure 4F).

### 2.5 Mechanistic Insights Into the Anti‐Glioma Effects of SB525334 Combined With CAR‐γδT Cells

**FIGURE 5:**
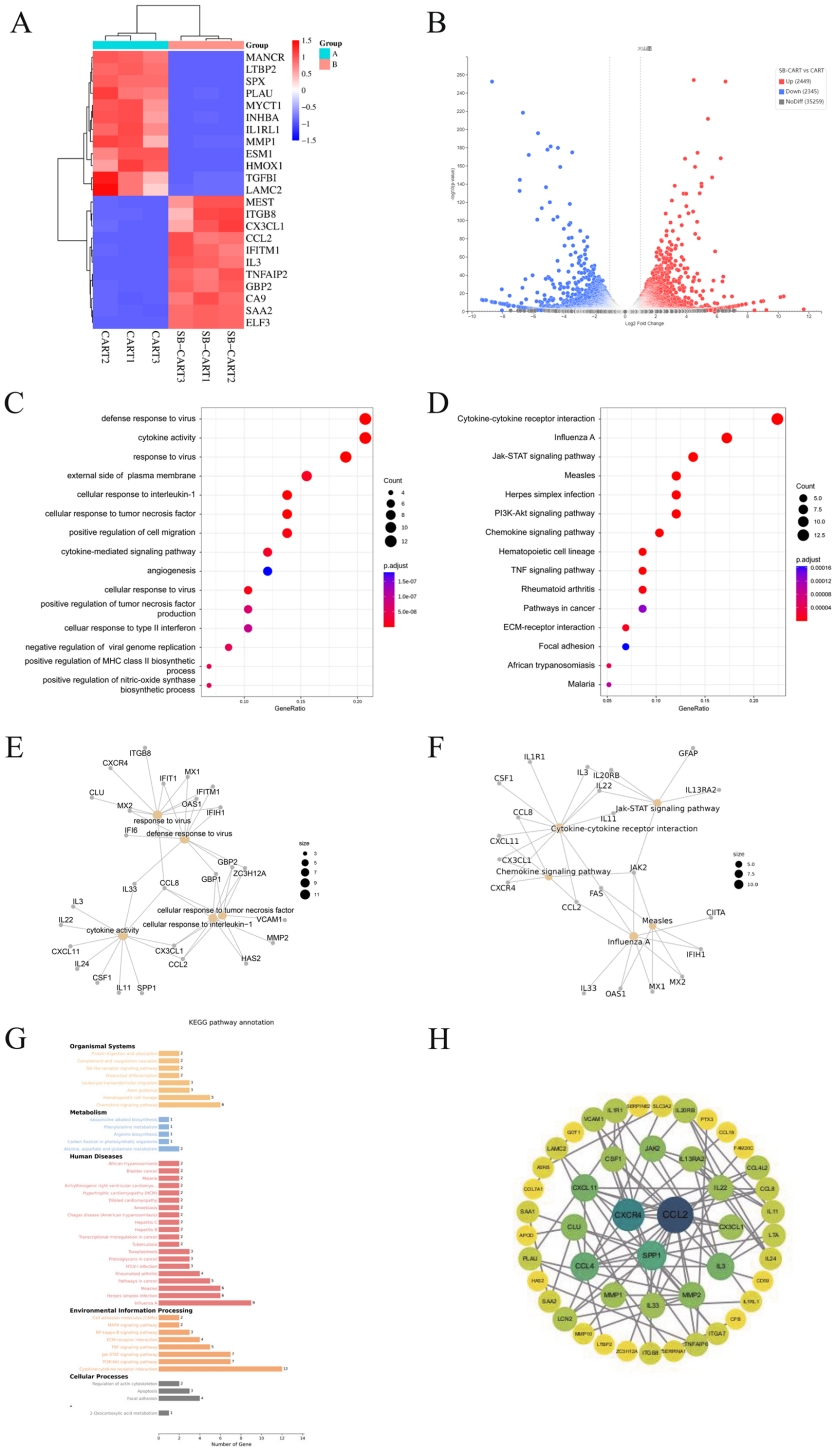
(A) Heatmap showing expression of key DEGs between the combination treatment group and the CART group. (B) Volcano plot of DEGs. (C, D) Bubble plots of GO and KEGG enrichment analyses, respectively. (E, F) Network diagrams of GO and KEGG enrichment. (G) KEGG pathway annotation. (H) Protein–protein interaction network analysis.

To elucidate the mechanism by which the TGF‐β receptor inhibitor SB525334 enhances the efficacy of CAR‐γδT cell therapy in glioma, RNA‐seq was performed to analyse differential gene expression profiles between the CAR‐γδT group (CAR T1–CAR T3) and the SB‐CAR‐γδT group (SB‐CAR T1–SB‐CAR T3). Heat map analysis revealed significant expression changes: genes such as LTBP2 and INHBA were downregulated (blue), whereas HMOX1 and IFNG were upregulated (red), with intensity validated by the scale (−1.5 to 1.5). Clustering analysis showed clear separation between groups, indicating altered transcriptional profiles (Figure 5A). A volcano plot quantified extensive expression differences, identifying 2449 upregulated and 2345 downregulated genes in the SB‐CAR T group versus the CAR T group, with log₂ fold changes and *p*‐value distributions reflecting statistical significance (Figure 5B).

Gene Ontology (GO) enrichment analysis, visualised by a bubble chart, revealed significant enrichment in immune‐ and antiviral‐related pathways (e.g., defence response to virus, cytokine activity), characterised by high gene counts and low adjusted *p*‐values (gene ratio ≈0.10) (Figure 5C). KEGG pathway analysis further indicated activation of cancer‐related and signalling pathways (e.g., Jak–STAT signalling, P13K–Akt signalling and extracellular matrix (ECM)–receptor interaction), with high enrichment (gene ratio 0.05–0.20) and low adjusted *p*‐values (Figure 5D). Network visualisation integrating GO and KEGG highlighted core pathways such as cytokine–cytokine receptor interaction and Jak–STAT signalling, centered on hub genes including CCL2, CCL8 and JAK2 (Figure 5E,F). Protein–protein interaction network analysis emphasised central roles for key proteins such as CCL2, CXCR4 and SPP1 (Figure 5H). Collectively, these results suggest that SB525334 combination therapy remodels immune‐ and antiviral‐related pathways, potentially driving enhanced antitumour efficacy.

## 3 Discussion

The pronounced overexpression of TGFB1 in GBM and its strong correlation with poor survival align with prior clinical observations that TGF‐β signalling drives immunosuppression and treatment resistance [20]. Our bioinformatic analysis reaffirms that TGFB1 is the dominant prognostic isoform within the TGF‐β family, consistent with studies highlighting its role in maintaining glioma stem cells and inducing T‐cell dysfunction [12, 21].

To enhance the efficacy of cellular immunotherapy or CAR‐T cells, multiple strategies targeting the TGF‐β pathway have been developed, encompassing genetic engineering approaches and small‐molecule inhibitors, as detailed below:

1. A novel inverted cytokine receptor, TB15, was designed that incorporates the extracellular domain of TGF‐βR2 for TGF‐β binding, a transmembrane region and the intracellular signalling domain of the pro‐inflammatory interleukin‐15 (IL‐15) receptor. TB15 converts immunosuppressive signals in the tumour microenvironment into cytokine‐mediated pro‐proliferative signals, thereby augmenting CAR‐T cell expansion and antitumour function [13].
2. Expression of the TGF‐β signalling suppressor SMAD7 in B7‐H3‐targeted CAR‐T cells.
3. Targeted degradation of SMAD2/SMAD3: Researchers developed synthetic substrate receptors (SySRs) using PROTAC technology, enabling either transgenic expression‐induced or small‐molecule‐dependent E3 ligase recruitment to degrade the transcription factors SMAD2/SMAD3, thereby blocking SMAD‐dependent TGF‐β signalling.
4. Small‐molecule inhibitors: For example, galunisertib, a TGF‐β type I receptor inhibitor, can be combined with neoadjuvant chemoradiotherapy.

Compared with other strategies, small‐molecule inhibitors offer greater simplicity and ease of use. Importantly, they may serve to remodel the tumour microenvironment. As a representative small‐molecule inhibitor, we evaluated the combination of SB525334 with CAR‐γδT cell therapy for glioma. Low‐dose SB525334 combined with CAR‐γδT cells significantly reduced tumour cell viability—achieving nearly 60% higher killing efficiency than CAR‐γδT treatment alone, demonstrating a synergistic effect. Unlike earlier agents that often caused systemic toxicity or nonspecific immune modulation, SB525334 preserved CAR‐γδT viability at therapeutic concentrations while potently inhibiting Smad2 phosphorylation, indicating selective action on the TGF‐β pathway without compromising T‐cell integrity [22, 23].

Notably, SB525334 delayed CAR‐γδT exhaustion during repeated antigen exposure, as evidenced by reduced exhaustion markers (LAG3, TIM3, CTLA4 and PD1) and sustained proliferative capacity. Our multiround stimulation assay showed that SB525334‐treated CAR‐γδT cells maintained superior cytotoxic function after multiple challenges, outperforming results from studies using PD‐1 blockade alone [24]. This suggests that targeting TGF‐β signalling may address a fundamental mechanism of T‐cell dysfunction that checkpoint inhibitors only partially reverse [10].

The in vivo results further substantiate the translational potential of this combination. SB525334 not only enhanced tumour regression and improved survival rates but also remodelled the glioma immune microenvironment by promoting effective T‐cell infiltration and suppressing multiple immunosuppressive cell populations. This addresses a critical hurdle noted in prior CAR‐γδT trials for GBM [25, 26]. The increased abundance of residual CAR‐γδT cells in the brains of combination‐treated mice aligns with evidence that TGF‐β inhibition can enhance T‐cell trafficking by modulating chemokine expression and overcoming stromal barriers [11]. Preconditioning the immune microenvironment represents a promising strategy to enhance the efficacy of cellular therapies and is increasingly being explored in the context of solid tumour immunotherapy [27]. Notably, the marked therapeutic effect observed in the SB pretreatment group (SB Pre–CAR T) further indicates that direct engineering of CAR‐γδT cells themselves constitutes a feasible and effective approach.

Another important consideration is the identification of biomarkers to predict response to the combination therapy. Our transcriptomic analysis showed that downregulation of LTBP2 and INHBA was associated with enhanced antitumour efficacy. These genes could serve as potential predictive biomarkers. For example, patients with baseline high expression of LTBP2 or INHBA may benefit more from SB525334‐mediated TGF‐β inhibition, as the therapy can effectively suppress these pro‐tumorigenic factors. In addition, monitoring changes in exhaustion markers (PD1, LAG3, TIM3, CTLA4) on circulating or tumour‐infiltrating CAR‐γδT cells during treatment could provide real‐time insight into treatment response and help guide dose adjustments. However, the clinical utility of these biomarkers needs validation in large patient cohorts, as preclinical models may not fully recapitulate the heterogeneity of human GBM.

The role of tumour stroma in GBM also warrants further investigation in the context of the SB525334‐CAR‐γδT combination. GBM stroma comprises various cell types, including cancer‐associated fibroblasts, endothelial cells and ECM components, which contribute to immunosuppression and therapy resistance. KEGG pathway analysis showed that SB525334 treatment affected ECM–receptor interaction, suggesting potential modulation of the stromal microenvironment. However, the specific mechanisms by which SB525334 alters stromal cell function and how these changes influence CAR‐γδT cell infiltration and activity remain unclear. Future studies could use single‐cell RNA sequencing to characterise changes in stromal cell populations after SB525334 treatment and investigate crosstalk between stromal cells and CAR‐γδT cells. This knowledge could enable additional strategies to target stroma and further improve the efficacy of the combination therapy.

Despite these advances, this study has several limitations. The use of immunodeficient mouse models may not fully recapitulate the complexity of human immune interactions, particularly the roles of regulatory T cells and myeloid populations in shaping the response to TGF‐β inhibition [8, 28]. In addition, although SB525334 showed favourable selectivity, its pharmacokinetics and blood–brain barrier penetration in humans require optimization. The focus on a single TGF‐β inhibitor also leaves open questions about whether dual targeting of other immunosuppressive pathways (e.g., IL‐10 or VEGF) could yield additive benefits [29]. Furthermore, antigen heterogeneity and loss—a common resistance mechanism in GBM—were not addressed here, suggesting that targeting TGF‐β alone may be insufficient for long‐term control without multiantigen targeting [30].

Finally, the safety profile of TGF‐β receptor inhibitors combined with CAR‐γδT therapy requires comprehensive evaluation in clinical trials. Although preclinical studies showed that SB525334 had minimal toxicity to CAR‐γδT cells at therapeutic concentrations, systemic inhibition of TGF‐β signalling may cause adverse effects in humans. TGF‐β has a critical role in normal tissue homeostasis, and long‐term inhibition could lead to complications such as fibrosis, autoimmune disease, or cardiovascular events. In clinical trials, close monitoring of adverse events, including immune‐related adverse events (irAEs) and organ function, will be essential. In addition, the timing and duration of SB525334 administration need optimization to balance efficacy and safety. For example, short‐term preconditioning with SB525334 before CAR‐γδT infusion may reduce the risk of long‐term toxicity while achieving the desired microenvironment remodelling.

Moreover, profiling dynamic changes in the tumour immune landscape during therapy will be essential to identify biomarkers of response and resistance [31]. Given the role of TGF‐β across multiple solid tumours, this strategy holds promise for broadening the applicability of CAR‐γδT therapy beyond GBM, particularly in TGF‐β–driven malignancies such as pancreatic or ovarian cancer [32].

## 4 Materials and Methods

### 4.1 Cell Lines

Human U251 and U87 cells (obtained from ATCC and confirmed mycoplasma‐free by PCR) were maintained in DMEM (GIBCO) supplemented with 10% (v/v) FBS (GIBCO), 100 U/mL penicillin (GIBCO) and streptomycin (GIBCO). All cells were cultured under standard conditions (37°C, 5% CO_2_).

### 4.2 CAR‐γδT Cells

This study used allogeneic anti‐B7H3 CAR‐γδT cells generated as previously described [18]. In brief, γδT cells were isolated from apheresis and collected from healthy donors (obtained from different individuals). After lentiviral transduction and expansion, CAR‐γδT cells were harvested and cryopreserved in liquid nitrogen before use.

### 4.3 SB525334 Treatment

SB525334 (Absin Bioscience, Cat. abs810374; dissolved in DMSO) was used. Stock solutions were diluted in culture medium immediately before use. The final DMSO concentration was maintained below 0.1% (v/v) in all experiments, including controls.

### 4.4 Bioinformatics Analysis of TGFB1 Expression and Prognosis

#### 4.4.1 Data Acquisition

TGFB1 expression in glioblastoma (GBM) and normal brain tissues was analysed using the GEPIA database (http://gepia.cancer‐pku.cn/). Independent validation was performed using transcriptomic data from public databases (TCGA and GTEx), which included 163 GBM tumour samples and 207 normal control samples.

#### 4.4.2 Expression Analysis

Differential expression of TGFB1 was evaluated. Box plots were generated to visualise TGFB1 expression, and a nonparametric Mann–Whitney *U* test was used to compare median TGFB1 expression levels between GBM and normal tissue groups. Statistical significance was defined as *p* < 0.001.

#### 4.4.3 Survival Analysis

Patients were stratified into TGFB1 high‐expression (*n* = 41) and low‐expression (*n* = 41) groups based on a predefined optimal cutoff. Kaplan–Meier survival curves were generated to compare overall survival (OS) and progression‐free survival (PFS) between groups, and the log‐rank test was used to assess statistical significance. A univariate Cox proportional hazards model was applied to calculate HRs and corresponding *p*‐values (*p* = 0.015 for OS; *p* = 0.05 for PFS).

#### 4.4.4 Prognostic Analysis of TGF‐β Isoforms

Prognostic associations were evaluated for all TGF‐β isoforms (TGFB1/ENSG00000105329.9, TGFB2/ENSG00000092969.11, TGFB3/ENSG00000119699.7). A heat map was generated to visualise log10 (HR) values derived from univariate Cox regression models.

### 4.5 In Vitro Evaluation of SB525334 Effects

#### 4.5.1 GBM Cell Viability Assay

U251 and U87 cells were seeded in 96‐well plates. After adherence, cells were treated with increasing concentrations of SB525334 (0, 5, 10, 20, 40, 80 μM) for 24, 48 and 72 h. Cell viability was assessed using the CCK‐8 kit (Absin Bioscience, Cat. abs50003) and expressed as a percentage relative to solvent‐treated control cells. Experiments were performed in triplicate.

#### 4.5.2 CAR‐γδT Cell Viability Assay

CAR‐γδT cells were seeded and treated with the same concentration gradient of SB525334 (0–80 μM) for 24, 48 and 72 h. Viability was evaluated using the CCK‐8 assay and reported as a percentage relative to untreated CAR‐γδT controls.

#### 4.5.3 Western Blot Analysis

U251 cells co‐treated with TGF‐β1 and 5 μM SB525334 for 2, 4, 6 and 12 h were lysed on ice using ice‐cold RIPA buffer. Protein concentrations were determined by BCA assay. Equal amounts of protein were separated by SDS–PAGE and transferred to PVDF membranes. After blocking with 5% skim milk, membranes were incubated with primary antibodies (anti‐phospho‐Smad2, ABclonal Technology, Cat. AP1342) overnight at 4°C. After washing with TBST, membranes were incubated with secondary antibodies for 1 h at room temperature. Signals were developed using an enhanced chemiluminescence (ECL) substrate and captured with a chemiluminescence imaging system.

### 4.6 In Vitro CAR‐γδT Functional Assays With SB525334

#### 4.6.1 Tumour Killing Assay

U251 or U87 cells were seeded in appropriate plates. TGF‐β1 (5 ng/mL) was added to mimic immunosuppressive conditions. Cells were co‐cultured for 48 h under the following conditions: medium control (NC), SB525334 alone (5 μM), CAR‐γδT cells alone at effector‐to‐target (E:T) ratios of 0.1:1 and 0.2:1, or CAR‐γδT cells combined with SB525334 at the same E:T ratios. Residual tumour cell viability was quantified using a luciferase‐based assay and reported as a percentage relative to the NC group.

#### 4.6.2 Migration Assay (Wound Healing)

U251 cells were grown to confluence in 24‐well plates. A scratch wound was created using a sterile pipette tip. Cells were treated with TGF‐β1 (5 ng/mL) alone or with TGF‐β1 plus increasing concentrations of SB525334. Migration into the wound area was monitored at defined time points (e.g., 0 and 24 h) by phase‐contrast microscopy and quantified with ImageJ.

#### 4.6.3 Transwell Migration Assay (Boyden Chamber)

Serum‐starved U251 cells were placed in the upper chamber of a Transwell insert (8 μm pore size), with the lower chamber containing medium supplemented with TGF‐β1 (5 ng/mL) with or without SB525334. After 48 h, migrated cells on the lower surface were fixed, stained and counted.

#### 4.6.4 CAR‐γδT Exhaustion and Functional Persistence (Repeated Stimulation)

A total of 2 × 10^5^ CAR‐γδT cells were co‐cultured with target U251 cells at an E:T ratio of 2:1 in medium with or without TGF‐β1 (5 ng/mL). SB525334 (5 μM) was added to the designated co‐treatment groups. After 48 h, when tumour cells were completely lysed, CAR‐γδT cells were counted, recovered by centrifugation and re‐stimulated with fresh tumour cells at the same E:T ratio. This process was repeated every 2 days until loss of killing capacity was observed, indicated by tumour cell growth.

#### 4.6.5 Exhaustion Marker Analysis (Flow Cytometry and qPCR)

After the final stimulation round, CAR‐γδT cells were collected. Surface expression of exhaustion markers (PD1, LAG3, TIM3, CTLA4) was analysed by flow cytometry using fluorophore‐conjugated antibodies. RNA was extracted from parallel samples, and mRNA levels of PDCD1 and IFNG were quantified by RT–qPCR and normalised to GAPDH.

### 4.7 In Vivo Antitumour Efficacy Study

#### 4.7.1 Mouse Models

All animal experiments were approved by the Institutional Animal Care and Use Committee (IACUC) of [Soochow University] and conducted in accordance with relevant guidelines.

##### 4.7.1.1 Orthotopic Tumour Model

Human GBM cells expressing luciferase (U251‐luc, 1 × 10^5^ cells) were intracranially implanted into immunodeficient mice (BALB/c Nude mice). Tumour growth was monitored by bioluminescence imaging (BLI) using an IVIS system after intraperitoneal (i.p.) injection of D‐luciferin (150 mg/kg).

##### 4.7.1.2 T‐Cell Infiltration Orthotopic Model

Mouse GBM cells (GL261, 5 × 10^4^ cells/2 μL) were intracranially implanted into immunocompetent C57BL/6 mice.

#### 4.7.2 Treatment Groups and Administration

##### 4.7.2.1 Efficacy Study (Nude Mice)

After confirming tumour establishment (approximately 7 days post‐implantation), tumour‐bearing mice were randomised into five groups (*n* = 6–9 per group):

- Group 1: NC—solvent control.
- Group 2: SB525334 alone (SB)—administered daily by oral gavage at 10 mg/kg starting on day 8.
- Group 3: CAR‐γδT alone (CART)—a single intracranial (IC) injection of CAR‐γδT cells (e.g., 5 × 10^4^ cells per mouse) on day 8; this group served to validate the therapeutic effect of the existing treatment.
- Group 4: SB525334 + CAR‐γδT combination (CART+SB) – SB525334 treatment (as in Group 2) combined with CAR‐γδT administration (as in Group 3). SB525334 treatment began before CAR‐γδT infusion.
- Group 5: SB525334‐pre‐treated CAR‐γδT (SB Pre‐CART) – CAR‐γδT cells were pre‐incubated with SB525334 (5 μM) in vitro for 6 h, thoroughly washed, and then administered via intracranial injection. No further SB525334 was administered following the infusion.

##### 4.7.2.2 T‐Cell Infiltration Study (C57BL/6 Mice)

Mice bearing intracranial GL261 tumours were randomised into two groups:

- Group 1: NC—solvent control.
- Group 2: SB525334 (SB)—administered as above for 2 weeks.

#### 4.7.3 Tumour Monitoring and Survival Analysis

BLI was performed weekly (days 7, 14, 21, 28 and 35 post‐implantation). Total flux (photons/s) was quantified for each animal. Survival was monitored daily until collective euthanasia on day 50. Kaplan–Meier survival curves were generated.

#### 4.7.4 T‐Cell Infiltration Assessment (C57BL/6 Model)

At the study endpoint, brains were harvested from GL261 tumour‐bearing C57BL/6 mice.

##### 4.7.4.1 Flow Cytometry

Single‐cell suspensions were prepared from enzymatically digested brain tumour tissue. Cells were stained with fluorophore‐conjugated antibodies (anti‐CD3). Tumour‐infiltrating lymphocytes were quantified as a percentage of total cells by flow cytometry.

##### 4.7.4.2 Immunofluorescence (IF)

Brain tissues were fixed, paraffin‐embedded and sectioned. Sections were stained with specific antibodies corresponding to the target cells and counterstained with DAPI. Images were acquired by confocal microscopy.

##### 4.7.4.3 Immunohistochemistry (IHC)

Brain tissues were fixed, embedded in paraffin, sectioned, deparaffinised and rehydrated. Antigen retrieval was performed in citrate buffer (pH 6.0) at 95°C for 20 min. Sections were incubated overnight at 4°C with primary antibodies against CD3 and Ki67, followed by HRP‐conjugated secondary antibodies, DAB development and haematoxylin counterstaining. Images were captured by light microscopy.

### 4.8 Transcriptomic Analysis

#### 4.8.1 Sample Preparation

RNA was isolated from the following in vitro groups under conditions mimicking the in vitro tumour killing assay: CAR‐γδT cells alone (CART; biological replicates CART1–CART3) and SB525334‐treated CAR‐γδT cells (SB‐CART; biological replicates SB‐CART1–SB‐CART3). High‐quality total RNA was extracted with TRIzol.

#### 4.8.2 RNA Sequencing

RNA‐seq libraries were prepared with the VAHTS Universal V10 RNA‐seq Library Prep Kit (Premined Version) and sequenced on the DNBSEQ‐T7 platform to generate paired‐end reads (2 × 150 bp).

#### 4.8.3 Bioinformatic Analysis

##### 4.8.3.1 Data Processing

Raw data were quality‐filtered using Trimmomatic to obtain clean data. Filtered reads were aligned to the reference genome using HISAT2 (v2.2.1) and quantified using StringTie.

##### 4.8.3.2 Differential Gene Expression Analysis

DESeq2 (v1.34.0) was used to identify differentially expressed genes (DEGs) between conditions. DEGs were initially filtered using |Fold Change| ≥ 2 and FDR < 0.05. If the number of DEGs exceeded 1000 per comparison, thresholds were tightened to |FC| ≥ 2 and FDR < 0.01. In this study, a false discovery rate (FDR) threshold of < 0.01 was applied to identify DEGs.

##### 4.8.3.3 Visualisation

Heatmaps of significantly dysregulated genes (e.g., LTBP2, INHBA, HMOX1) were generated from *z* scores of normalised counts. Volcano plots displayed all genes by log2FC and adjusted *p* values.

##### 4.8.3.4 Functional Enrichment Analysis

DEG lists were analysed for GO enrichment (biological process, molecular function, cellular component) and KEGG pathway enrichment. Significance was defined as FDR < 0.05. Results were visualised with bubble plots showing gene ratio, enrichment score and P value. Protein–protein interaction (PPI) networks for key functional clusters were explored using STRING and visualised in Cytoscape. Core modules and hub genes were identified.

### 4.9 Statistical Analysis

Statistical analyses were conducted in GraphPad Prism (v10.4.1). Data are reported as mean ± SEM. Two‐group differences were evaluated with the unpaired Student *t*‐test for parametric data or the Mann–Whitney *U* test for nonparametric data. Multiple‐group comparisons were assessed with one‐way or two‐way ANOVA, as appropriate. Survival was analysed with the log‐rank (Mantel–Cox) test. Statistical significance was set at *p* < 0.05, *p* < 0.01, or *p* < 0.001. All in vitro experiments included at least three biological replicates.

## Author Contributions

Conception and design: Y.Z., Z.H., X.L. Development of methodology: Y.Z., Z.H., Z.Z., Y.S., Y.Z., W.W., Z.J. Analysis and interpretation of data: Y.Z., Q.E., X.Z., Y.H., J.D., Y.T., H.D., Y.X. Writing, review, and/or revision of the manuscript: Y.Z., R.S., C.L. Administrative, technical, or material support: Y.H., X.L. Guarantor: Y.H.

## Funding

This study was supported by the National Natural Science Foundation of China (82173279 and 82472981), National Science and Technology Resource Sharing Service Platform Project (YCZYPT [2020]06‐1), Suzhou Medical and Health Innovation Project (CXYJ2024A05), Gusu Talent Program (2024) 105, Suzhou Industrial Park Healthcare Talent Support Initiative (2024) 54 and Clinical Priority Disease Diagnosis and Treatment Program (LCZX202347). The study sponsors did not influence the study design, the collection, analysis and interpretation of the data, and had no role in the writing of the manuscript.

## Conflicts of Interest

The authors declare no conflicts of interest or personal relationships that could have influenced the work reported in this paper. Beijing Unicet Biotechnology Co. Ltd. provided the CAR‐γδT cells but had no role in study design, data collection and analysis, decision to publish, or manuscript preparation.

## Supporting information


**Table S1.** Glossary of tumour type abbreviations used in this study.

## Data Availability

Data supporting the findings of this study are available from the corresponding author upon request. The data are not publicly available due to privacy and ethical considerations.
