# Supplementary material for: TGF‐β Blockade With SB525334 Enhances B7‐H3 CAR‐γδT Cell Efficacy Against Glioblastoma
Source: J Cell Mol Med. 2026 Mar 25;30(6):e71089. doi: 10.1111/jcmm.71089 (PMC13097692; doi:10.1111/jcmm.71089)
Supplement: Supplementary file 1 — Table S1. Glossary of tumour type abbreviations used in this study. [file JCMM-30-e71089-s001.docx]

**Glossary of Tumor Type Abbreviations Used in This Study**

| **Abbreviation** | **Full Name (Common Cancer Name)** |
| --- | --- |
| **ACC**​ | Adrenocortical carcinoma |
| **BRCA**​ | Breast invasive carcinoma |
| **CESC**​ | Cervical squamous cell carcinoma and endocervical adenocarcinoma |
| **CHOL**​ | Cholangiocarcinoma |
| **DLBC**​ | Lymphoid Neoplasm Diffuse Large B-cell Lymphoma |
| **ESCA**​ | Esophageal carcinoma |
| **GBM**​ | Glioblastoma multiforme |
| **HNSC**​ | Head and Neck squamous cell carcinoma |
| **KICH**​ | Kidney Chromophobe |
| **KIRP**​ | Kidney renal papillary cell carcinoma |
| **LGG**​ | Brain Lower Grade Glioma |
| **LIHC**​ | Liver hepatocellular carcinoma |
| **LUAD**​ | Lung adenocarcinoma |
| **LUSC**​ | Lung squamous cell carcinoma |
| **OV**​ | Ovarian serous cystadenocarcinoma |
| **PAAD**​ | Pancreatic adenocarcinoma |
| **PCPG**​ | Pheochromocytoma and Paraganglioma |
| **READ**​ | Rectum adenocarcinoma |
| **SARC**​ | Sarcoma |
| **SKCM**​ | Skin Cutaneous Melanoma |
| **TGCT**​ | Testicular Germ Cell Tumors |
| **THCA**​ | Thyroid carcinoma |
| **THYM**​ | Thymoma |
| **UCEC**​ | Uterine Corpus Endometrial Carcinoma |
| **UCS**​ | Uterine Carcinosarcoma |
